# Utility of Flow Cytometry for Prognostic Prediction in Adult T‐Cell Leukemia/Lymphoma

**DOI:** 10.1002/jha2.70345

**Published:** 2026-07-03

**Authors:** Shigeo Fuji, Masahito Tokunaga, Lucy Cook, Atae Utsunomiya, Junya Makiyama, Youko Suehiro, Ki‐Ryang Koh, Makoto Nakashima, Yoshihisa Yamano, Kaoru Uchimaru

**Affiliations:** ^1^ Department of Hematology Osaka International Cancer Institute Osaka Japan; ^2^ Department of Hematology Imamura General Hospital Kagoshima Japan; ^3^ National Centre for Human Retrovirology and Department of Haematology Imperial College Healthcare NHS Trust London UK; ^4^ Department of Hematology Sasebo City General Hospital Nagasaki Japan; ^5^ Department of Hematology and Cell Therapy National Hospital Organization Kyushu Cancer Center Fukuoka Japan; ^6^ Department of Hematology Osaka General Hospital of West Japan Railway Company Osaka Japan; ^7^ Laboratory of Tumor Cell Biology Department of Computational Biology and Medical Sciences Graduate School of Frontier Sciences University of Tokyo Tokyo Japan; ^8^ Department of Neurology St. Marianna University School of Medicine Kanagawa Japan

**Keywords:** adult T‐cell leukemia/lymphoma, CD4 count, flow cytometry, human T‐lymphotropic virus type 1, indolent, prognostic factor

## Abstract

**Introduction:**

Adult T‐cell leukemia/lymphoma (ATL) exhibits marked clinical heterogeneity; however, current classification primarily rely on morphology and laboratory findings and require further advancement. This study aimed to evaluate the roles of human T‐lymphotropic virus type 1 (HTLV‐1)–infected cell (CD4‐positive T cell) analysis system using flow cytometry (HAS‐Flow) and absolute CD4 count in prognostic assessment of nonaggressive ATL.

**Methods:**

We retrospectively analyzed 227 individuals with HTLV‐1 infection in Japan (derivation cohort) and 81 in the United Kingdom (validation cohort). HAS‐Flow categorized patients into G1–G4 groups according to the proportion of cell adhesion molecule 1 (CADM1)^+^/CD7^−^ cells. Clinical outcomes were compared by HAS‐Flow and absolute CD4 count (cutoff: 1500 cells/µL) using Kaplan–Meier and Cox analyses.

**Results:**

In the derivation cohort, 4‐year progression‐free survival (PFS) rates were 100% for G1–G2, 94.2% for G3, and 55.7% for G4 (*p* < 0.01). Absolute CD4 counts >1500 cells/µL identified patients with significantly shorter PFS and overall survival. G4 patients showed poor prognosis irrespective of CD4 counts. The UK validation cohort reproduced the prognostic CD4 counts >1500 cells/µL.

**Conclusion:**

HAS‐Flow and absolute CD4 count provide additive prognostic information beyond the Shimoyama classification.

**Trial Registration:**

The authors have confirmed clinical trial registration is not needed for this submission.

## 1 Introduction

Adult T‐cell leukemia/lymphoma (ATL) is a peripheral T‐cell malignancy caused by the infection of human T‐lymphotropic virus type 1 (HTLV‐1), primarily affecting CD4‐positive T cells [1, 2]. Aggressive ATL, such as acute and lymphoma subtypes, has an extremely poor prognosis [1]. The disease is endemic in southwestern Japan, the Caribbean basin, parts of Africa, and South America, with an estimated 10–20 million HTLV‐1 carriers worldwide [3]. However, there has been an increasing number of cases in developed countries, likely reflecting migration from endemic regions [4, 5, 6].

The clinical heterogeneity of ATL has posed significant challenges in diagnosis, prognostication, and treatment selection [2, 3]. In 1991, Shimoyama proposed a landmark classification system of clinical subtypes based on a comprehensive analysis of 813 ATL cases by the Japan Clinical Oncology Group‐Lymphoma Study Group (JCOG‐LSG) [7]. This classification stratifies ATL into four distinct clinical subtypes: Acute, lymphoma, chronic, and smoldering types, based on peripheral blood lymphocyte count, percentage of abnormal lymphocytes, presence of flower cells, lactate dehydrogenase (LDH) levels, corrected calcium values, and organ involvement patterns. The Shimoyama classification has been widely adopted internationally and has remained the cornerstone of ATL diagnosis and treatment decision‐making for over three decades.

Despite its clinical utility, the Shimoyama classification has inherent limitations in prognostic stratification. The classification primarily relies on conventional laboratory parameters and morphological features, since modern diagnostic methods, such as multicolor flow cytometry, were unavailable in clinical practice 40 years ago [7]. Particularly, it would be practically difficult to achieve consistent agreement in the determination of a small number of abnormal lymphocytes in peripheral blood [8, 9, 10]. Consequently, the reproducibility of clinical classification based solely on morphology is limited [8, 10]. Moreover, patients with the same subtype often exhibit considerable variation in clinical outcomes, suggesting that additional biomarkers are needed to refine prognostic accuracy, which could lead to a more sophisticated treatment strategy, including possible preventive measures to reduce the risk of progression to acute‐type ATL in high‐risk patients [9, 11, 12, 13].

Recent advances in flow cytometry technology have transformed diagnostic and response assessment criteria across several hematologic malignancies, such as chronic lymphocytic leukemia (CLL), T‐large granular lymphocytic leukemia (T‐LGL), and multiple myeloma (MM) [14, 15]. Flow cytometry has also enabled the development of sophisticated analytical methods for HTLV‐1‐infected cells. The HTLV‐1‐infected cell analysis system using flow cytometry (HAS‐Flow) represents a novel approach that focuses on the expression patterns of cell adhesion molecule 1 (CADM1) and CD7 on CD4‐positive T cells [9, 12, 13, 16]. The downregulation of CD7 expression has been associated with clonal expansion and disease progression in HTLV‐1 carriers [16]. Previous studies have demonstrated that the proportion of CADM1‐positive CD4 cells, particularly those with CD7 downregulation, correlates with HTLV‐1 proviral load (PVL) and may serve as a predictor of ATL development [9, 12, 13]. These findings suggest that HAS‐Flow may capture important biological features of ATL pathogenesis that are not reflected in the Shimoyama classification. However, research in this area remains limited.

This study aimed to conduct a retrospective analysis to assess the prognostic impact of HAS‐Flow in addition to clinical subtype and other prognostic markers in HTLV‐1‐infected individuals.

## 2 Patients and Methods

This retrospective study included two datasets for derivation and validation. The derivation dataset included 227 individuals with HTLV‐1 infection who were treated at the Research Hospital, the Institute of Medical Science, the University of Tokyo (IMSUT), or the Imamura General Hospital. The indolent ATL database was built from the Joint Study on Predisposing Factors of ATL Development (JSPFAD) program. The validation dataset included 81 individuals with HTLV‐1 infection who were treated at Imperial College London. For UK patients, written informed consent was obtained from all participants, and the study was approved by the UK National Research Ethics Service (09/H0606/106, 15/SC/0089, 20/SC/0226, 25/SC/0209). Clinical subtypes were classified into HTLV‐1 carrier or ATL according to the Shimoyama classification at the time of first flow cytometry analysis [2, 7]. Patients with acute or lymphoma‐type ATL and those lacking follow‐up were excluded. In terms of the smoldering‐type ATL, patients with skin lesions were excluded. Peripheral blood samples were collected from individuals with HTLV‐1 infection from whom written informed consent was obtained according to the procedures approved by the institutional review board (IRB). This study was approved by the IRB at the Osaka International Cancer Institute (No. 21210). The study was conducted in accordance with the Declaration of Helsinki.

The HAS‐Flow method was described previously [9, 12, 13, 16, 17]. Briefly, the HAS‐Flow method creates a CADM1 versus CD7 plot of CD4‐positive T cells, allowing for the identification of distinct cellular populations: P population (CADM1‐negative, CD7‐positive), D population (CADM1‐positive, CD7‐positive～dim positive), and N population (CADM1‐positive, CD7‐negative). A representative HAS‐Flow analysis is shown in Figure S1. The antibodies used in the HAS‐Flow method are listed in Table S1. Following prior reports, cases were categorized into four groups according to the proportion of cells in the D and N regions: G1 (D + N ≤ 10%), G2 (10% < D + N ≤ 25%), G3 (25% < D + N ≤ 50%), and G4 (50% < D + N) [13, 16]. We added the grouping of HAS‐Flow to the clinical subtypes of Shimoyama's criteria, and categorized individuals with HTLV‐1 infection into groups as follows: HTLV‐1 carrier with HAS G1 or G2 (G1–G2 carrier), HTLV‐1 carrier with G3 (G3 carrier), smoldering‐type ATL with G3 (G3 smoldering), smoldering‐type ATL with G4 (G4 smoldering), chronic‐type ATL with G3 (G3 chronic), and chronic‐type ATL with G4 (G4 chronic).

In terms of data analysis for absolute CD4 count, datasets from Japanese institutes were used for the derivation group. We evaluated the association between absolute lymphocyte count (ALC) and absolute CD4+ T‐cell count (CD4) using both a simple linear regression model and a piecewise linear regression model with a pre‐specified breakpoint at 1500 cells/µL, which is the upper limit of normal of absolute CD4 count. For the piecewise model, a hinge term was defined as max (0, CD4–1500), allowing a change in slope above the breakpoint. Model fit was compared between the two approaches using a likelihood ratio test (LRT), Akaike information criterion corrected for small sample size (AICc), and 10‐fold cross‐validation with root mean square error (RMSE) as the performance metric. For the validation group, we obtained data of individuals with HTLV‐1 infection at Imperial College London.

The probabilities of overall survival (OS) and progression‐free survival (PFS) were computed from the survival curves estimated by the Kaplan–Meier method, and differences between the groups were assessed using the log‐rank test. The definition of PFS in this study was the introduction of systemic chemotherapy, progression to acute or lymphoma‐type ATL, or death. We also analyzed ATL‐specific PFS by treating death by causes other than ATL as a censor. The Cox proportional‐hazards regression model was used for OS analysis in multivariate analyses. For multivariate analyses, HAS‐grouping (G1–G2 vs. G3 vs. G4), PVL (continuous), and CD4 count (low vs. high) were included, and no stepwise selection was performed. The correlation between two parameters was assessed using Spearman's rank test. A *p*‐value of < 0.05 indicates statistical significance.

All statistical analyses were performed with EZR (Jichi Medical University Saitama Medical Center, Saitama, Japan; http:// www.jichi.ac.jp/saitama‐sct/SaitamaHP.files/statmedEN.html), a graphical user interface for R version 1.54 (The R Foundation for Statistical Computing, Vienna, Austria) [18]. More precisely, EZR is a modified version of R Commander version 2.7–1 that incorporates widely used packages, including survival and cmprsk.

## 3 Results

Between 2009 and 2024, 227 individuals with HTLV‐1 infection at Japanese institutes were enrolled in this study. The patient characteristics were grouped according to HAS‐Flow and clinical subtypes (Table 1). The median age was 63 years (range, 29–90). The median follow‐up of surviving patients was 1639 days (range, 14–5465).

**TABLE 1:** Baseline characteristics of patients according to human T‐lymphotropic virus type 1‐infected cell analysis system using flow cytometry grouping and clinical subtype.

		Group							
Factor		G1 carrier	G2 carrier	G3 carrier	G3 smoldering	G3 chronic	G4 smoldering	G4 chronic	*p* value
*n*		48	55	30	32	1	26	35	
Age	Median (range)	61.5 [35, 84]	64 [29, 80]	63 [36, 77]	61 [35, 78]	50	64 [34, 90]	64 [35, 87]	0.253
Sex	Male	11 (22.9)	18 (32.7)	14 (46.7)	9 (28.1)	1 (100.0)	8 (30.8)	12 (34.3)	0.305
	Female	37 (77.1)	37 (67.3)	16 (53.3)	22 (68.8)	0 (0.0)	16 (61.5)	21 (60.0)	
	NA	0 (0.0)	0 (0.0)	0 (0.0)	1 (3.1)	0 (0.0)	2 (7.7)	2 (5.7)	
PVL (copies/100 PBMC)	Median (range)	1.98 [0.01, 12.1]	6.79 [0.77, 23.3]	12.9 [4.1, 24.7]	18.3 [7.0, 40.3]	18.06	33.2 [9.2, 144.5]	56.8 [1.6, 266.1]	<0.001
soluble IL‐2 receptor (U/mL)	Median (range)	333 [196, 1100]	408 [165, 1540]	501.5 [232, 1400]	555 [328, 1580]	562	993.5 [372, 13900]	1930 [507, 11300]	<0.001
Total lymphocyte count (µL)	Median (range)	1509 [390, 3917]	1618 [385, 3819]	1678 [655, 4041]	2113 [841, 3459]	5687	2886 [591, 3853]	6469 [4026, 13996]	<0.001
Total CD4 count (µL)	Median (range)	645 [52, 1614]	685 [107, 2260]	771 [242, 1796]	795 [47, 1875]	2906	1496 [363, 2771]	4055 [1154, 11419]	<0.001
	≤1500	45 (93.8)	49 (89.1)	29 (96.7)	25 (78.1)	0 (0.0)	13 (50.0)	2 (5.7)	<0.001
	>1500	1 (2.1)	2 (3.6)	1 (3.3)	4 (12.5)	1 (100.0)	13 (50.0)	32 (91.4)	
	NA	2 (4.2)	4 (7.3)	0 (0.0)	3 (9.4)	0 (0.0)	0 (0.0)	1 (2.9)	
HAS‐flow (D+N) (%)	Median (range)	4.8 [0.6, 10.0]	16.0 [10.1, 24.8]	33.0 [25.3, 50.0]	37.4 [25.6, 49.9]	38.2	64.9 [51.9, 87.6]	87.2 [51.0, 97.5]	<0.001

*Note*: Baseline demographic and clinical features of individuals with HTLV‐1 infection (*n* = 232, derivation cohort). Patients were stratified according to HAS‐Flow groups (G1–G2, G3, G4) and clinical subtypes (carrier, smoldering, chronic). Values are presented as median (range) for continuous variables and as number (percentage) for categorical variables.

Abbreviations: D+N, D population (CADM1‐positive, CD7‐positive), and N population (CADM1‐positive, CD7‐negative); HAS‐Flow, human T‐lymphotropic virus type 1‐infected cell analysis system using flow cytometry; HTLV‐1, human T‐lymphotropic virus type 1; NA, not available; PBMC, peripheral blood mononuclear cells; PVL, proviral load.

**FIGURE 1:**
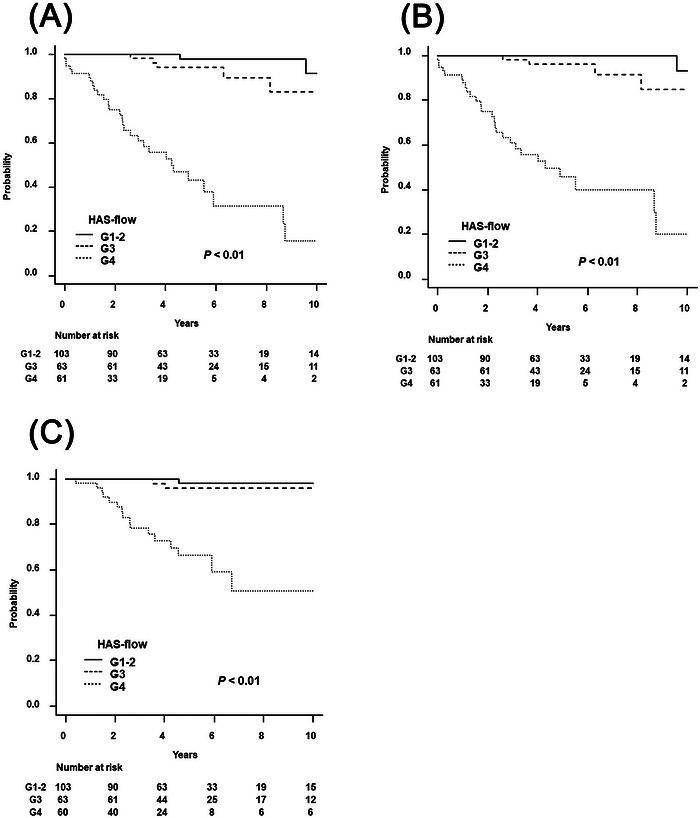
Kaplan–Meier estimates of progression‐free and overall survival according to human T‐lymphotropic virus type 1‐infected cell analysis system using flow cytometry grouping. (A) Progression‐free survival (PFS). (B) ATL‐specific PFS. (C) Overall survival (OS) in patients stratified by HAS‐Flow grouping (G1–G2, G3, G4) in the derivation cohort (*n* = 232). *p*‐values were calculated using the log‐rank test. HAS‐Flow, human T‐lymphotropic virus type 1‐infected cell analysis system using flow cytometry; ATL, adult T‐cell leukemia/lymphoma.

The probabilities of 4‐year PFS were 100% in the G1–G2 group, 94.2% (95% confidence interval [CI], 83.0–98.1) in the G3 group, 55.7% (95% CI, 40.0–68.9) in the G4 group (*p* < 0.01, Figure 1A). The probabilities of 4‐year PFS were 100% in G1 carrier, 100% in G2 carrier, 100% in G3 carrier, 88.5% (95% CI, 68.3–96.2) in G3 smoldering, 69.5% (95% CI, 45.9–84.4) in G4 smoldering, and 45.8% (95% CI, 25.7–63.8) in G4 chronic (*p* < 0.01) (Figure S2A). No statistically significant difference was observed between G4 smoldering and G4 chronic.

The probabilities of 4‐year ATL‐specific PFS were 100% in the G1–G2 group, 96.2% (95% CI, 85.6–99.0) in the G3 group, and 55.7% (95% CI, 40.0–68.9) in the G4 group (*p* < 0.01) (Figure 1B). The probabilities of 4‐year ATL‐specific PFS were 100% in G1 carrier, 100% in G2 carrier, 100% in G3 carrier, 92.4% (95% CI, 72.5–98.0) in G3 smoldering, 69.5% (95% CI, 44.9–84.4) in G4 smoldering, and 45.8% (95% CI, 25.7–63.8) in G4 chronic (*p* < 0.01) (Figure S2B).

The probabilities of 4‐year OS were 100% in the G1–G2 group, 95.8% (95% CI, 84.2–98.9) in the G3 group, 72.6% (95% CI, 56.5–83.6) in the G4 group (*p* < 0.01) (Figure 1C). The probabilities of 4‐year OS were 100% in G1 carrier, 100% in G2 carrier, 100% in G3 carrier, 91.6% (95% CI, 70.4–97.8) in G3 smoldering, 79.6% (95% CI, 53.4–92.0) in G4 smoldering, and 67.1% (95% CI, 44.5–82.1) in G4 chronic (*p* < 0.01) (Figure S2C). Regarding OS rates, no statistically significant difference was observed between G3 carrier and G3 smoldering, and between G4 smoldering and G4 chronic.

**FIGURE 2:**
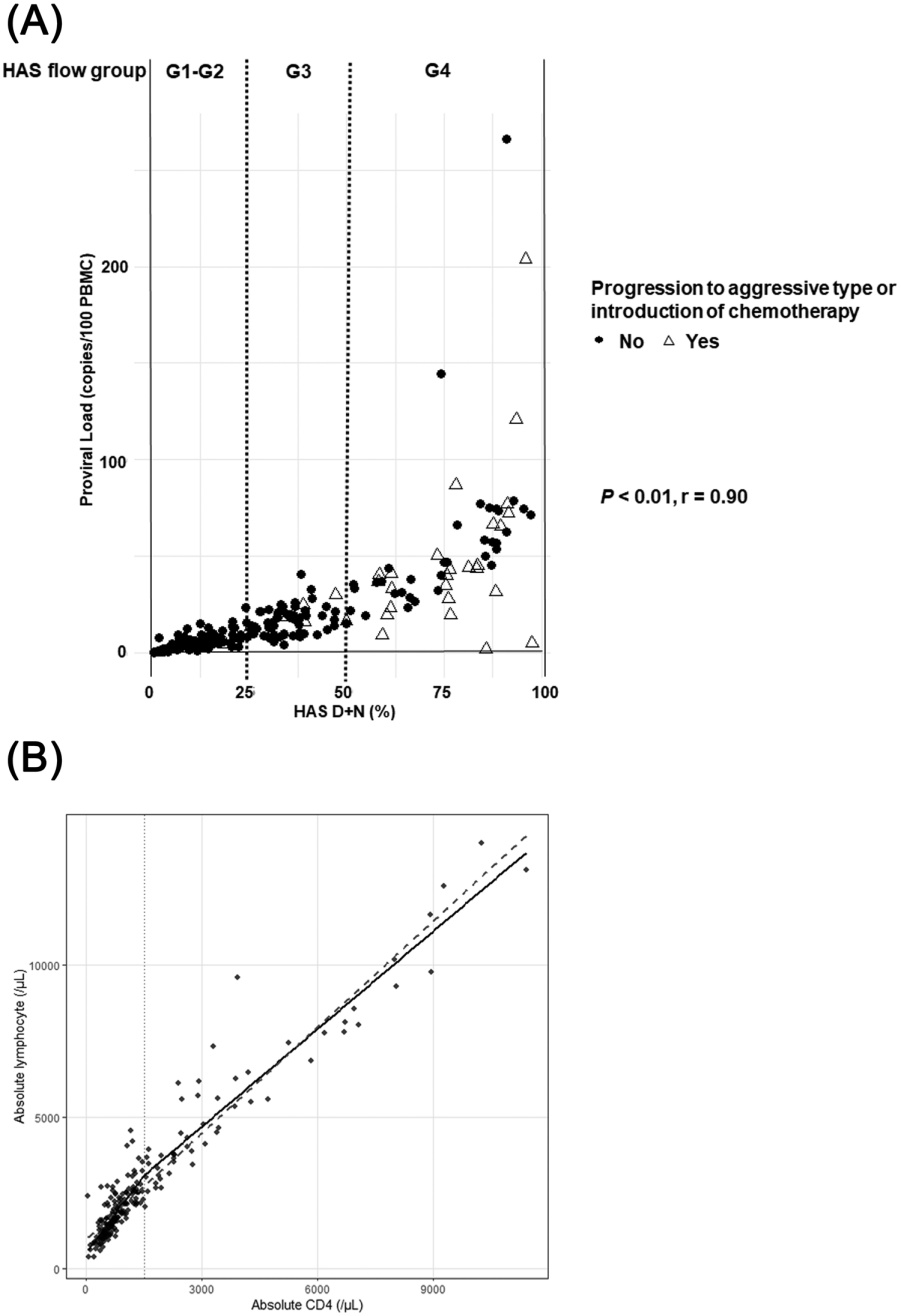
Association between human T‐lymphotropic virus type 1‐infected cell analysis system using flow cytometry pattern, proviral load, and disease progression. (A) Correlation between HAS‐Flow groups and HTLV‐1 proviral load (*r* = 0.90, *p* < 0.01). Patients who experienced progression or required chemotherapy are indicated by triangles. (B) Correlation between absolute CD4 count and absolute lymphocyte count. HAS‐Flow, human T‐lymphotropic virus type 1‐infected cell analysis system using flow cytometry.

PVL correlated significantly with HAS‐Flow patterns (*r* = 0.90, *p* < 0.01) (Figure 2A). We also assessed the association of these parameters with the progression or introduction of chemotherapy (Figure 2A). In our cohort, patients who experienced the progression or introduction of chemotherapy were mostly in G4 (shown as a triangle).

**FIGURE 3:**
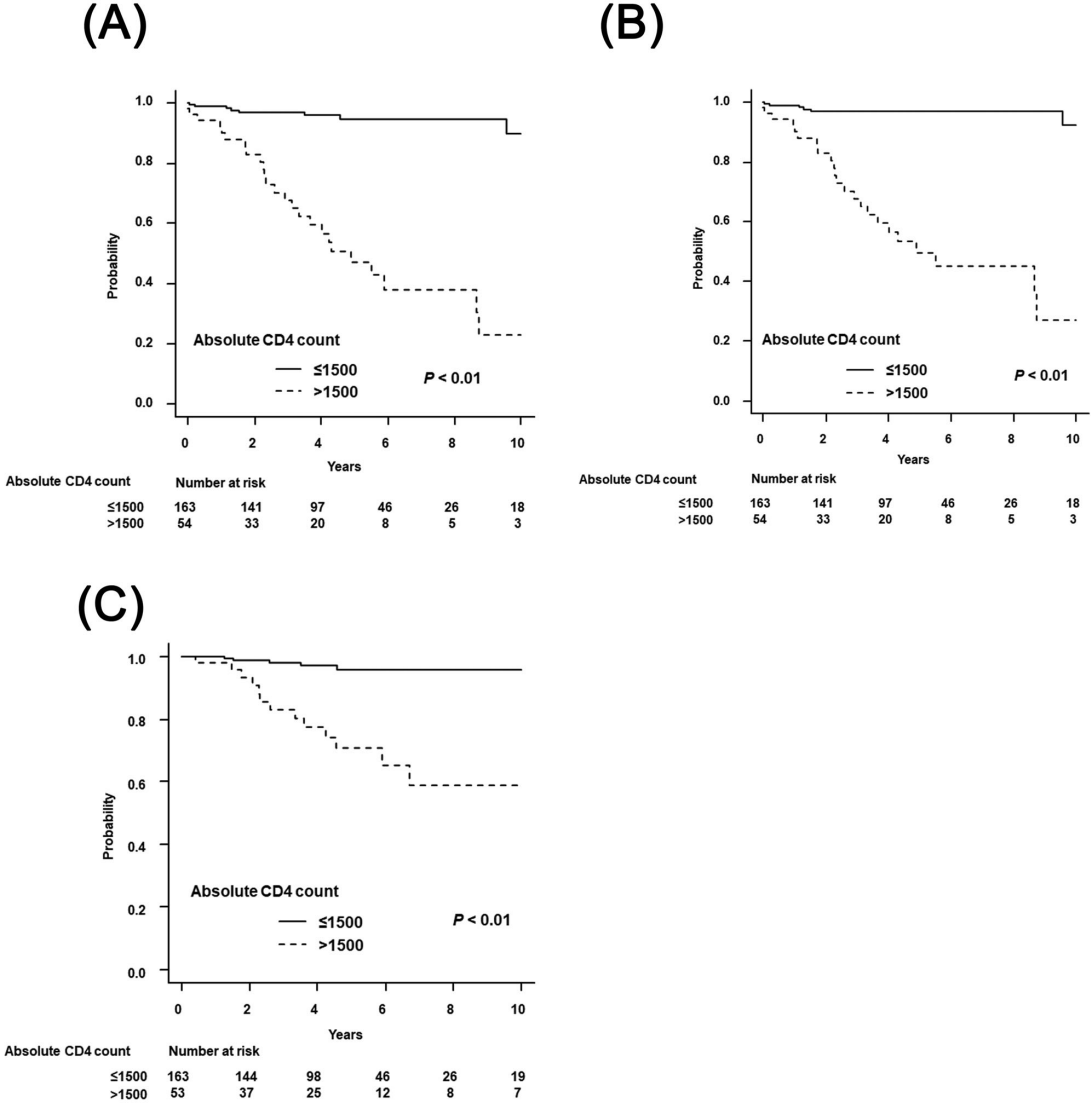
Prognostic impact of absolute CD 4 count in the derivation cohort. Kaplan–Meier curves of (A) progression‐free survival (PFS), (B) ATL‐specific PFS, and (C) overall survival (OS) in patients stratified by absolute CD4 count (≤1500 vs. >1500 cells/µL). *p*‐values were calculated using the log‐rank test. ATL, adult T‐cell leukemia/lymphoma.

As shown in Table 1, a significant association was observed between the grouping by HAS‐flow and absolute CD4 count, as well as ALC (Figure 2B). The piecewise linear regression model with a fixed breakpoint at the upper limit of normal (CD4, 1500 cells/µL) demonstrated a significantly better fit compared with the simple linear regression model (LRT: F = 23.1, *p* < 0.01). The AICc was substantially lower for the piecewise model than for the linear model (3426.2 vs. 3446.4) in favor of the piecewise specification. In addition, predictive performance was slightly improved in the piecewise model, with a lower mean RMSE on 10‐fold cross‐validation compared with the linear model (630.2 vs. 660.7). These findings support the hypothesis that the relationship between CD4 and total lymphocyte count changes at approximately 1500 cells/µL, consistent with the biologically plausible threshold, as 1500 cells/µL is the upper limit of normal. Accordingly, we divided patients into two groups (high CD4 count group and low CD4 count group). In the G4 smoldering and G4 chronic groups, the percentages of high CD4 count were 50.0% and 91.4%, respectively. A significant difference was observed in PFS, ATL‐specific PFS, and OS between the two groups (Figures 3A–C). We assessed the prognostic significance of HAS‐Flow in addition to CD4 count. We divided patients into four groups according to the combination of CD4 count (high CD4 count vs. low CD4 count) and HAS‐Flow (G1–G3 vs. G4). Although the number of cases with low CD4 count and HAS‐flow G4 group was limited, those cases had a similarly poor clinical outcome as in cases with high CD4 count and HAS‐flow G4 group (Figure S3A–C). We also assessed the association of PVL and CD4 count (r = 0.90, *p* < 0.01) (Figure S4). Furthermore, we assessed the association of these parameters with the progression or introduction of chemotherapy (Figure S4). In our cohort, patients who experienced the progression or introduction of chemotherapy were mostly in high CD4 count (shown as a triangle). We performed the multivariate analyses for PFS, ATL‐specific PFS, and OS, and found that HAS grouping and high CD4 count were independent prognostic factors for PFS and ATL‐specific PFS (Table S2).

**FIGURE 4:**
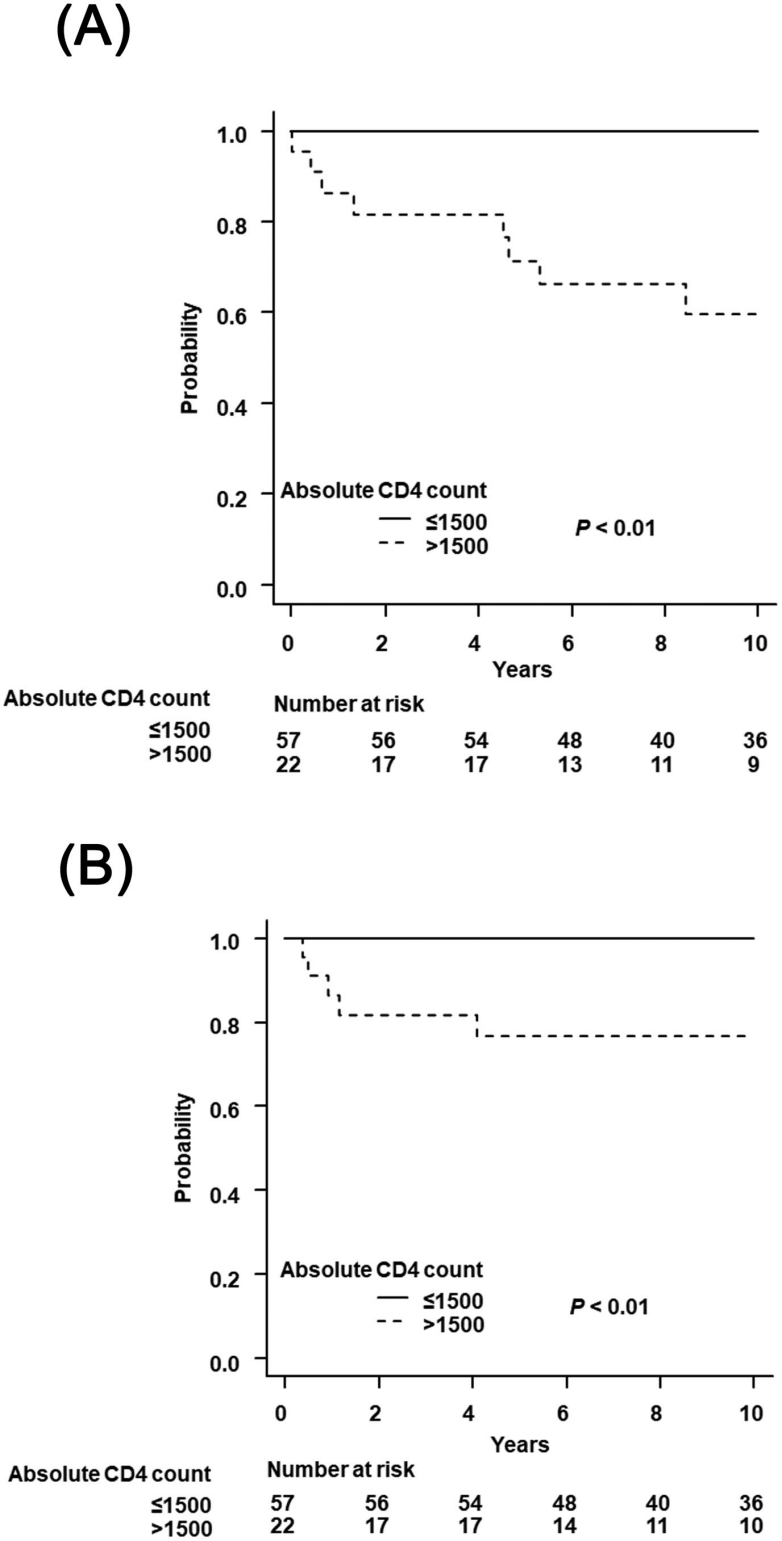
Prognostic impact of absolute CD4 count in the validation cohort. Kaplan–Meier curves of (A) progression‐free survival (PFS) and (B) overall survival (OS) in patients stratified by absolute CD4 count (≤1500 vs. >1500 cells/µL) in the validation dataset (Imperial College London).

To further confirm our findings, we used an independent dataset from Imperial College London. This validation dataset included 81 cases (HTLV‐1 asymptomatic carrier, *n* = 54; smoldering, *n* = 10; chronic, *n* = 17). The median age was 48 years (range, 17–83). After excluding two patients without available CD4 count data, we divided 79 patients into two groups (high CD4 count group and normal CD4 count group). A significant difference was observed in the PFS and OS between the two groups (Figure 4A,B).

## 4 Discussion

This study represents the most comprehensive assessment to date of the prognostic utility of flow cytometry in individuals with HTLV‐1 infection. To our knowledge, this is the largest cohort analyzed to date in individuals with HTLV‐1 infection, with flow cytometry data and a reasonable median follow‐up duration. Our findings indicate that HAS‐Flow analysis provides a promising approach for prognostic stratification. It was noteworthy that similar overall clinical outcomes were observed between G3 carrier and G3 smoldering without skin lesions and between G4 smoldering and G4 chronic, with important implications of flow cytometry for clinical practice compared to classical clinical subtypes of Shimoyama's criteria, as the clinical subtypes did not have an additive effect to improve the prognostic prediction; that is, G3 carrier and G3 smoldering without skin lesions, and G4 smoldering and G4 chronic are indistinguishable [12, 13].

The HAS‐Flow grading system grouped individuals with HTLV‐1 infection into four groups, based on the proportion of CADM1‐positive/CD7‐negative cell populations [13, 16]. In our dataset, the progression rates to aggressive ATL in G1 and G2 were very low, with less than 5% over 5 years. In contrast, G4 had a progression rate of around 50%. It is intriguing that G3 also had a low progression rate over 5 years. However, the follow‐up period of around 4 years in our study may still be insufficient to assess progression risk in this population, as some events occurred beyond 5 years in this cohort.

We identified that absolute CD4 count could be a reliable prognostic marker in individuals with HTLV‐1 infection, which was demonstrated in the Japanese and UK cohorts. In Shimoyama's criteria, we used total lymphocyte count as a threshold; a total lymphocyte count ≥ 4000 cells/µL is required for the chronic type [7]. Given that ATL is a malignancy of CD4‐derived T‐cells proliferating in peripheral blood, it is reasonable that absolute CD4 count would reflect whether the status that ATL cells have already been in a proliferative phase, particularly when the CD4 count exceeds the upper limit of the normal value of 1500 cells.

We assessed the clinical significance of HAS‐Flow‐determined grouping, in addition to CD4 count. Although the proportion of cases with high CD4 counts in the HAS‐Flow G4 group was high, some cases still had low CD4 counts. We found that the clinical outcomes of these cases with low CD4 counts were comparable to those with high CD4 counts in the HAS‐Flow G4 group, suggesting that HAS‐Flow could identify patients at high risk even in the group with low CD4 counts. However, due to a limited sample size, the clinical significance of HAS‐Flow G4 with low CD4 count has to be reassessed in the future. When available, HAS‐Flow could be a preferable method to be performed in individuals with HTLV‐1 infection. However, in the setting of limited resources, CD4 count could be a reasonable method with low cost.

Importantly, absolute CD4 counts can be widely used worldwide, as they have been used for decades in the assessment of HIV infection. Furthermore, the cost and accessibility of the test are crucial for monitoring the disease status worldwide. Measuring absolute CD4 counts is considerably less expensive than modern multicolor flow cytometry or other advanced methods, such as clonality analysis, and does not require a costly flow cytometer. Given the clear prognostic value of absolute CD4 count in our two datasets and its widespread availability worldwide, it is reasonable to incorporate absolute CD4 count into the assessment of disease status in individuals with HTLV‐1 infection. In our cohort, most chronic‐type patients had elevated absolute CD4 counts, although some smoldering‐type patients also showed increased CD4 counts. Thus, the absolute CD4 count could more precisely reflect ATL cell proliferation in peripheral blood than the total lymphocyte count. Although the prognostic value of absolute CD4 count should be further evaluated in future studies, it could be incorporated into the definition of ATL clinical subtypes. Since a majority of chronic type ATL cases had CD4 counts of >1500 cells/µL, it may be reasonable to include CD4 counts of >1500 cells/µL in the definition of chronic type ATL.

This study has some limitations. First, the retrospective design and data from a few major centers limit the generalizability of our findings. Although we analyzed two independent datasets from Japan and the United Kingdom, we believe that our findings have to be validated in larger, multicenter prospective studies with a longer follow‐up period. Although our dataset is the largest to date with flow cytometry data from individuals with HTLV‐1 infection, the relatively small sample size, while adequate for demonstrating statistical significance, may not fully capture the biological diversity of ATL. Second, the median follow‐up period, while sufficient for PFS analysis, may be inadequate for comprehensive OS assessment in indolent disease subtypes. Third, we have to emphasize that each method has its own strengths and weaknesses. For instance, in our dataset, there were a few cases of chronic‐type ATL with very low PVL. In these cases, it was reported that they had deficient HTLV‐1 virus variants, which were undetectable by conventional PVL testing. Soluble interleukin‐2 receptor level, lymphocyte count, and HAS‐Flow could also be affected by various clinical factors, such as infectious diseases. Thus, it would be crucial to confirm the status of individuals with HTLV‐1 infection using multiple modalities to improve prognostication. Finally, the dynamic change of HAS‐Flow pattern could further improve the prognostic value of HAS‐Flow. Although the present study focused on the prognostic value of single‐point HAS‐Flow, HAS‐Flow was analyzed at several points according to disease progression in several cases and showed marked changes in the pattern. A representative case in which HTLV‐1 carrier status progressed to chronic ATL is shown in Figure S5. The value of longitudinal analysis using HAS‐Flow must be determined in future studies.

In conclusion, this study provided compelling evidence for the additive prognostic capability of flow cytometry and absolute CD4 count to the Shimoyama classification. The findings of this study could help develop strategies to advance ATL risk assessment and management, ultimately improving outcomes for patients with this challenging malignancy. The implementation of these biomarkers into clinical practice guidelines, as reflected in the revision of Shimoyama's criteria, represents an important step toward a modernized, precision‐medicine approach in the management of individuals with HTLV‐1 infection.

## Author Contributions

S.F., M.T., A.U., J.M., and K.U. designed the study. S.F., M.T., L.C., A.U., J.M., Y.S., KR.K., M.N., and Y.Y. collected clinical data. S.F. and K.U. analyzed clinical data. All authors reviewed and approved the final manuscript.

## Ethics Statement

For UK patients, the study was approved by the UK National Research Ethics Service (09/H0606/106, 15/SC/0089, 20/SC/0226, 25/SC/0209). For Japanese patients, this study was approved by the IRB at the Osaka International Cancer Institute (No. 21210).

## Consent

For UK patients, written informed consent was obtained from all participants. For Japanese patients, written informed consent was obtained from all participants.

## Conflicts of Interest

The authors declare no conflicts of interest.

## Supporting information




**Supplemental Table 1**. List of antibodies for HAS‐Flow

**Supplemental Figure 1**. A representative result of HAS‐Flow method

## Data Availability

Data are available from the corresponding author upon reasonable request.
