# Supplementary material for: Utility of Flow Cytometry for Prognostic Prediction in Adult T‐Cell Leukemia/Lymphoma
Source: EJHaem. 2026 Jul 3;7(4):e70345. doi: 10.1002/jha2.70345 (PMC13329845; doi:10.1002/jha2.70345)
Supplement: Supplementary file 2 — Supplemental Figure 1. A representative result of HAS‐Flow method [file JHA2-7-e70345-s001.pdf]

Supplemental Figure 1

Supplemental Figure 1. A representative result of HAS-Flow method

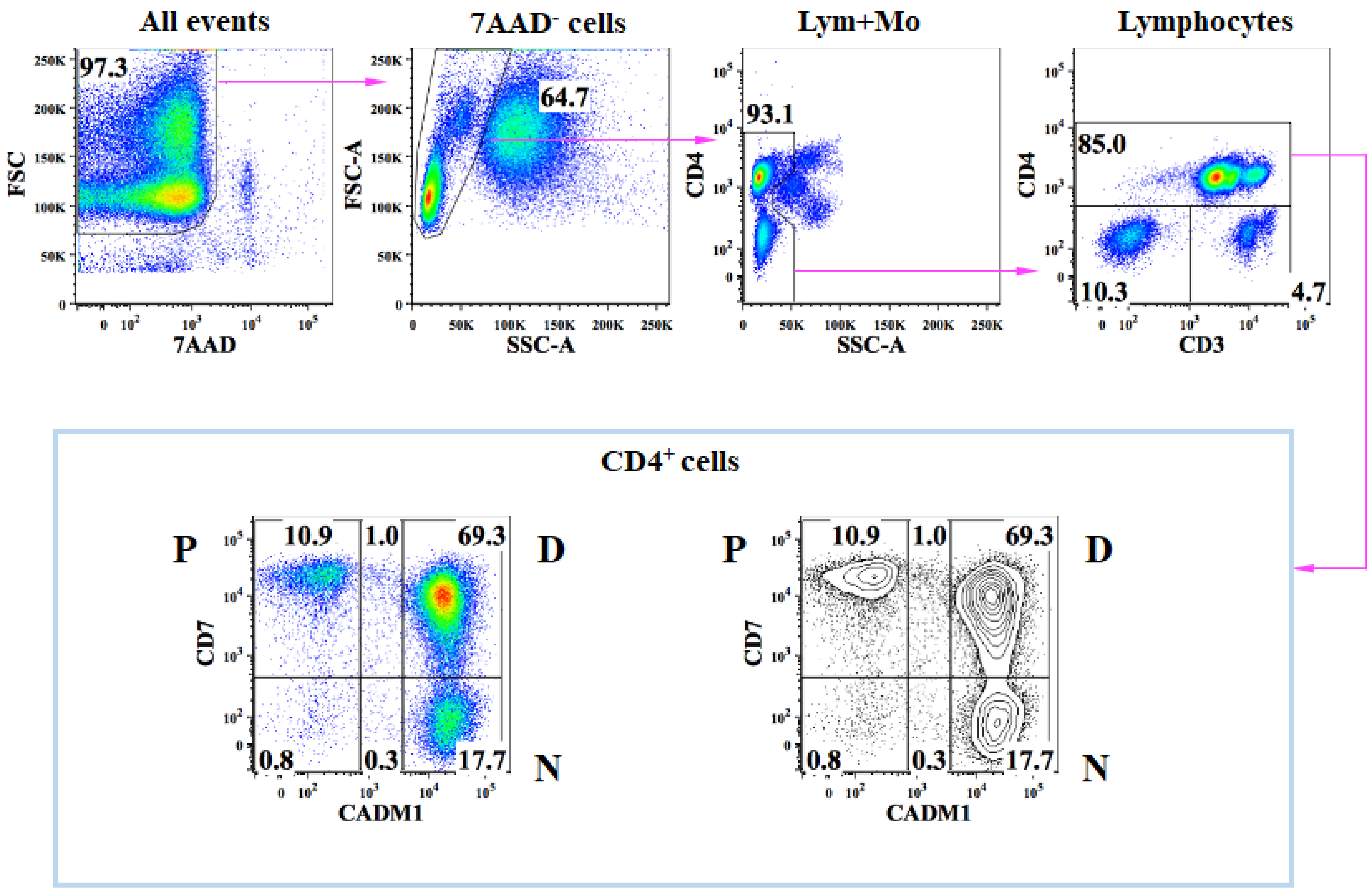

Supplemental Figure 2

(A)

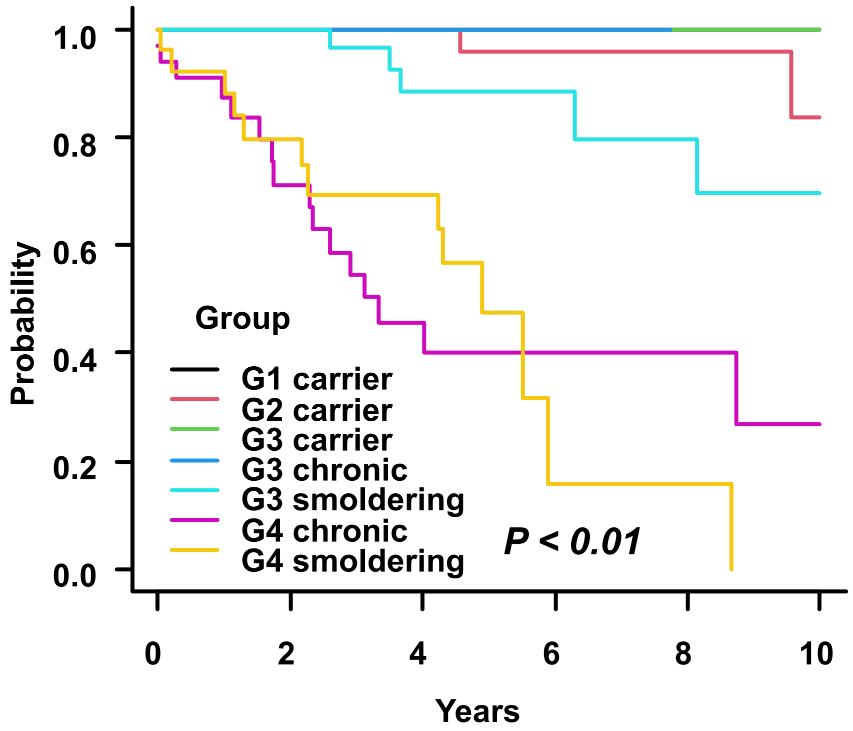

Group	Number at risk					
G1 carrier	48	41	32	16	11	7
G2 carrier	55	49	31	17	8	7
G3 carrier	30	30	22	12	7	6
G3 chronic	1	1	1	1	0	0
G3 smoldering	32	30	20	11	8	5
G4 chronic	35	17	8	4	3	2
G4 smoldering	26	16	11	1	1	0

Supplemental Figure 2. Kaplan–Meier estimates of survival by combined human T-lymphotropic virus type 1-infected cell analysis system using flow cytometry and clinical subtype.

PFS (A), ATL-specific PFS (B), and overall survival (C) according to the combined classification of HAS-Flow (G1–G4) and clinical subtype (carrier, smoldering, chronic) were evaluated. HAS-Flow, human T-lymphotropic virus type 1-infected cell analysis system using flow cytometry; ATL, adult T-cell leukemia/lymphoma; PFS, progression-free survival.

Supplemental Figure 2

(B)

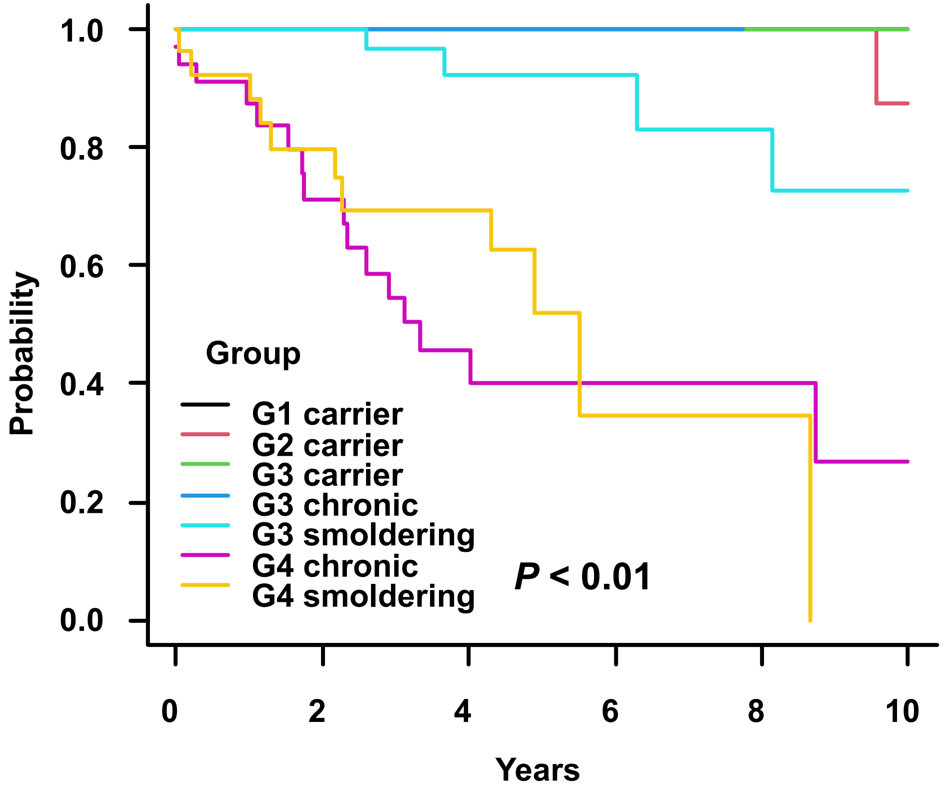

Number at risk

Group	0	2	4	6	8	10
G1 carrier	48	41	32	16	11	7
G2 carrier	55	49	31	17	8	7
G3 carrier	30	30	22	12	7	6
G3 chronic	1	1	1	1	0	0
G3 smoldering	32	30	20	11	8	5
G4 chronic	35	17	8	4	3	2
G4 smoldering	26	16	11	1	1	0

Supplemental Figure 2

(C)

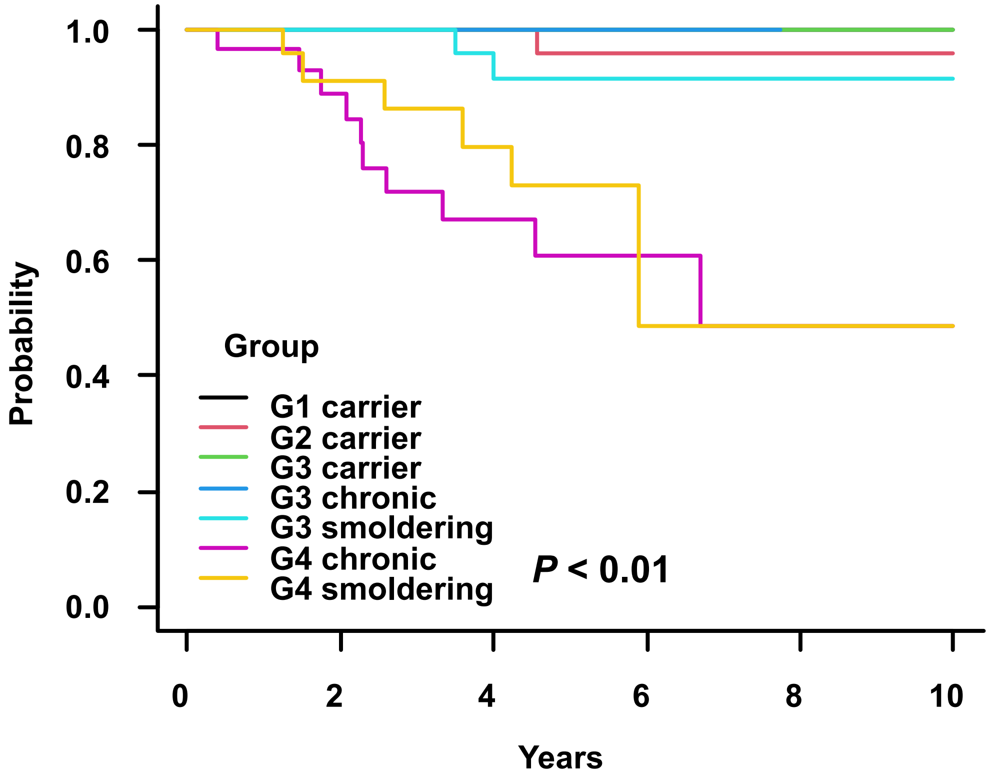

Group	Number at risk					
G1 carrier	48	41	32	16	11	7
G2 carrier	55	49	31	17	8	8
G3 carrier	30	30	22	12	7	6
G3 chronic	1	1	1	1	0	0
G3 smoldering	32	30	21	12	10	6
G4 chronic	35	21	12	6	4	4
G4 smoldering	25	19	12	2	2	2

Supplemental Figure 3

(A)

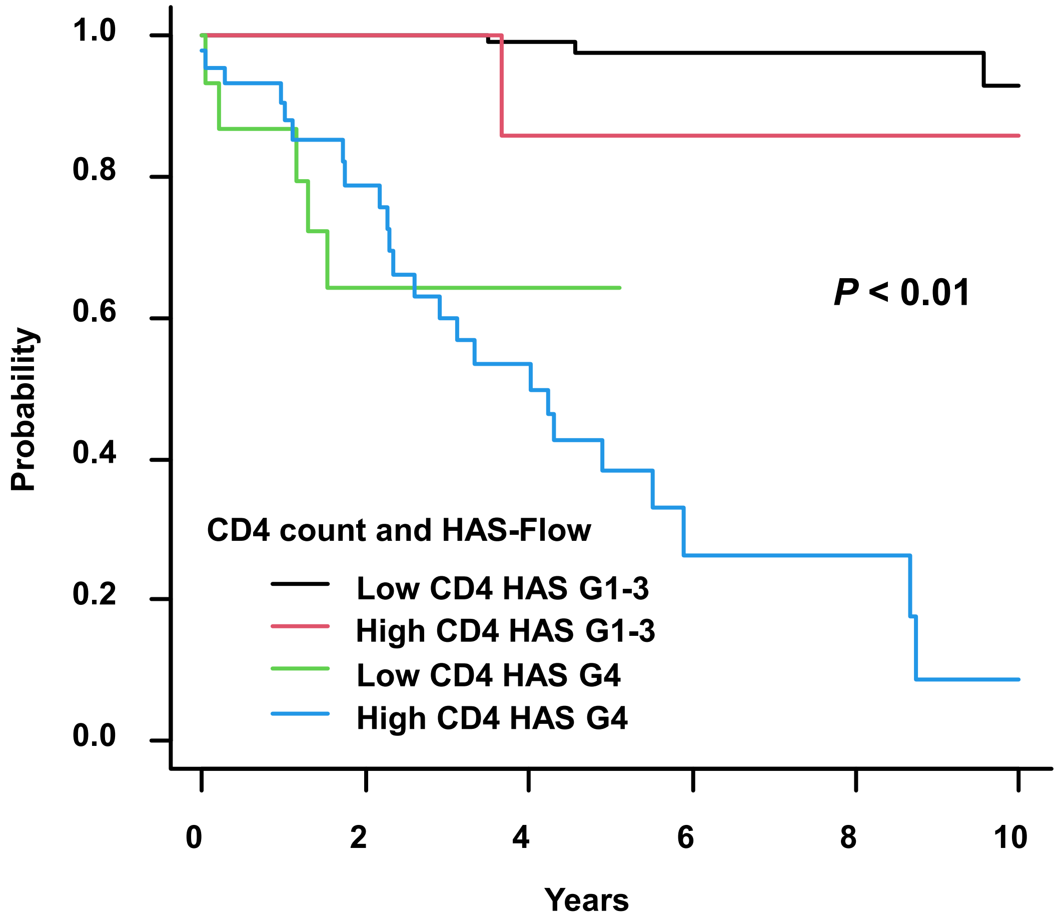

CD4 count and HAS-Flow	Number at risk					
Low CD4 HAS G1-3	148	134	94	46	26	18
High CD4 HAS G1-3	9	8	5	4	2	2
Low CD4 HAS G4	15	7	3	0	0	0
High CD4 HAS G4	45	25	15	4	3	1

Supplemental Figure 3. Kaplan–Meier estimates of survival by combined human T-lymphotropic virus type 1-infected cell analysis system using flow cytometry and CD4 count.

PFS (A), ATL-specific PFS (B), and overall survival (C) according to combined classification of HAS-Flow (G1-3 vs. G4) and CD4 count (low CD4 count vs. high CD4 count). HAS-Flow, human T-lymphotropic virus type 1-infected cell analysis system using flow cytometry; CD4, cluster of differentiation 4; PFS, progression-free survival.

Supplemental Figure 3

(B)

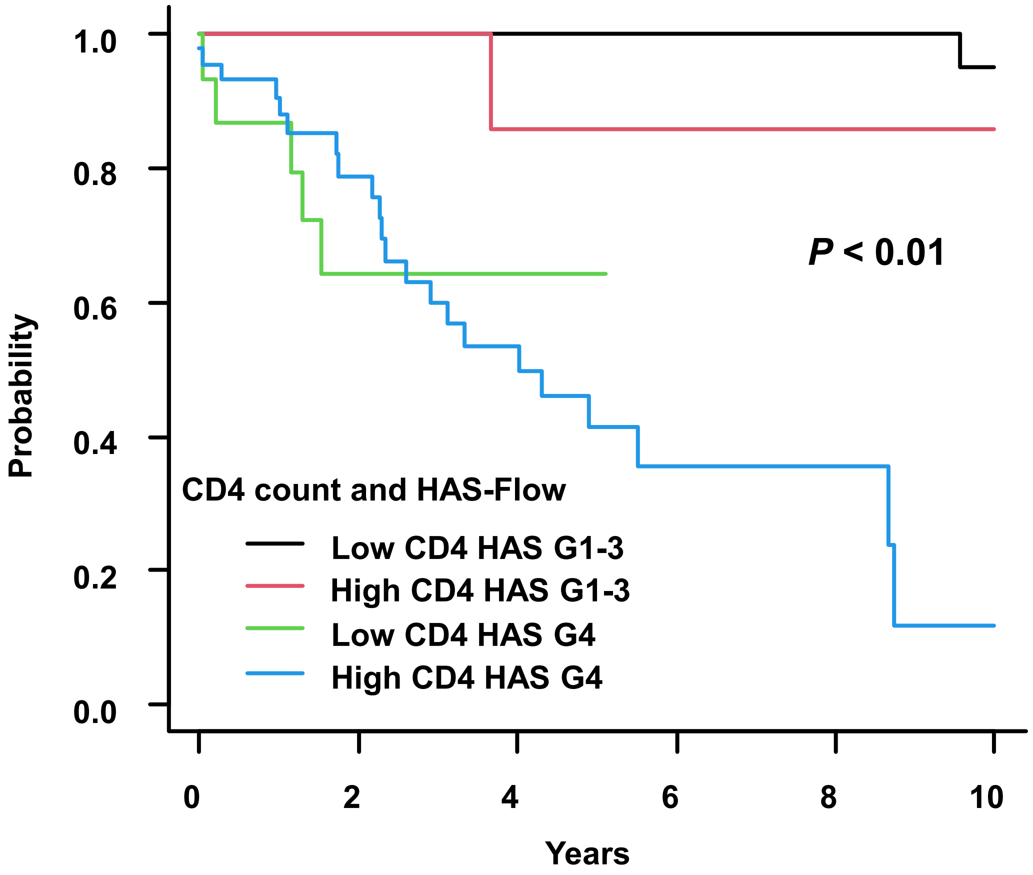

CD4 count and HAS-Flow	Number at risk					
Low CD4 HAS G1-3	148	134	94	46	26	18
High CD4 HAS G1-3	9	8	5	4	2	2
Low CD4 HAS G4	15	7	3	0	0	0
High CD4 HAS G4	45	25	15	4	3	1

Supplemental Figure 3

(C)

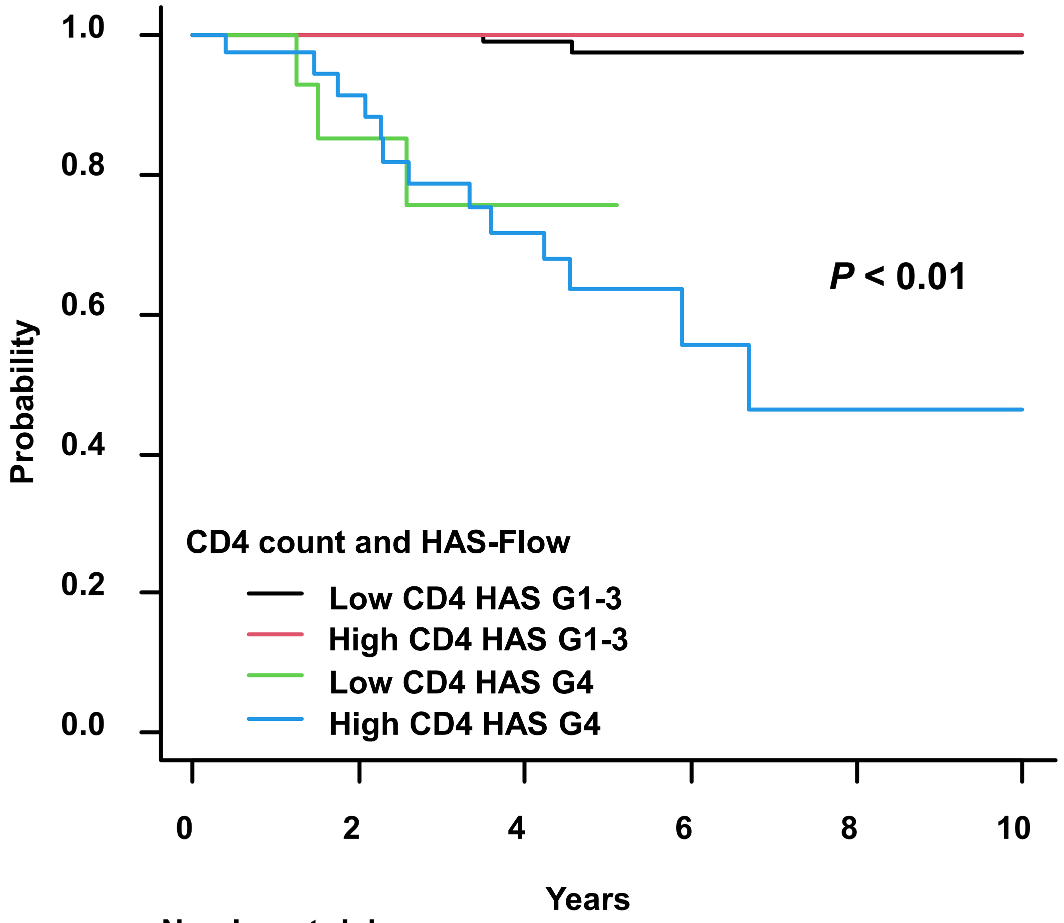

CD4 count and HAS-Flow	Number at risk					
Low CD4 HAS G1-3	148	134	94	46	26	19
High CD4 HAS G1-3	9	8	6	5	3	2
Low CD4 HAS G4	15	10	4	0	0	0
High CD4 HAS G4	44	29	19	7	5	5

Supplemental Figure 4

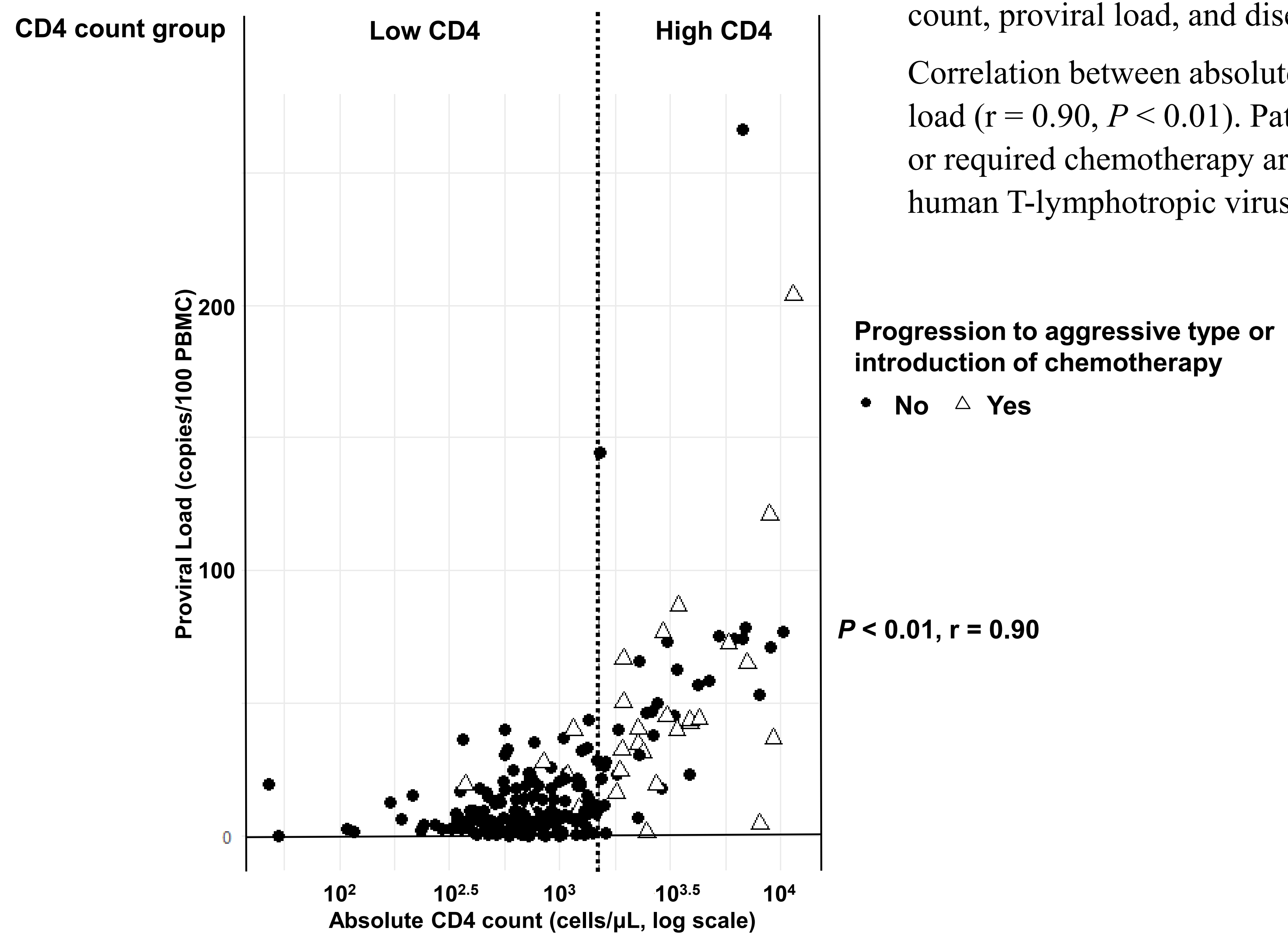

Supplemental Figure 4. Association between absolute CD4 count, proviral load, and disease progression.

Correlation between absolute CD4 count and HTLV-1 proviral load (r = 0.90, $P < 0.01$). Patients who experienced progression or required chemotherapy are indicated by triangles. HTLV-1, human T-lymphotropic virus type 1.

Supplemental Figure 5

Supplemental Figure 5. A case of ATL progression from HTLV-1 carrier status to chronic ATL

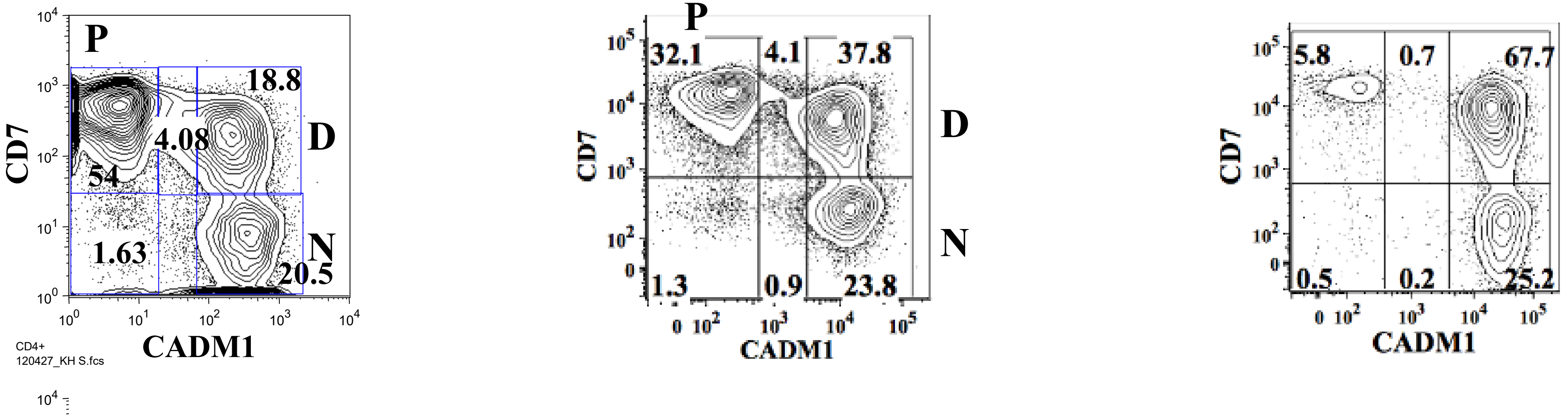

2012.4 AC PVL 9.77%   2019.4 smoldering PVL 32.78%   2023.10 chronic PVL 69.50%
